# Sex-dimorphic role of prefrontal oxytocin receptors in social-induced facilitation of extinction in juvenile rats

**DOI:** 10.1038/s41398-020-01040-9

**Published:** 2020-10-19

**Authors:** Mouna Maroun, Amit Sarussi-Elyahu, Aseel Yaseen, Ossama A. Hatoum, Milly Kritman

**Affiliations:** 1 Sagol Department of Neurobiology, Faculty of Natural Sciences, University of Haifa, Haifa, Israel; 2 Department of Surgery B- HaEmek Medical Center, Faculty of Medicine, Technion: Israel Institute of Technology, Afula, Israel

**Keywords:** Physiology, Learning and memory

## Abstract

We previously reported that in the adult animal extinction in pairs resulted in enhanced extinction, showing that social presence can reduce previously acquired fear responses. Based on our findings that juvenile and adult animals differ in the mechanisms of extinction, here we address whether the social presence of a conspecific affects extinction in juvenile animals similarly to adults. We further address whether such presence has a different impact on juvenile males and females. To that end, we examined in our established experimental setting whether conditioned male and female animals extinguish contextual fear memory better while in pairs. Taking advantage of the role of oxytocin (OT) in the mediation of extinction memory and social interaction, we also study the effect of antagonizing the OT receptors (OTR) either systemically or in the prefrontal cortex on social interaction-induced effects of fear extinction. The results show that social presence accelerates extinction in males and females as compared to the single condition. Yet, we show differential and opposing effects of an OTR antagonist in both sexes. Whereas in females, the systemic application of an OTR antagonist is associated with impaired extinction, it is associated with enhanced extinction in males. In contrast, prefrontal OT is not engaged in extinction in juvenile males, while is it is critical in females. Previously reported differences in the levels of prefrontal OT between males and females might explain the differences in OT action. These results suggest that even during the juvenile period, critical mechanisms are differently involved in the regulation of fear in males and females.

## Introduction

Fear memory can be weakened through extinction training^1–4^. Important open questions regarding extinction include which factors can modulate extinction and whether extinction can be enhanced without pharmacological intervention. We previously showed that fear extinction could be facilitated by extinction in pairs; i.e., two animals that undergo extinction together^5^. We further demonstrated that this phenomenon is dependent on oxytocin (OT) in the infralimbic subregion of the prefrontal cortex (IL-mPFC) since the microinfusion of an OT receptor (OTR) antagonist inhibited the extinction enhancement^5^. We previously showed that distinct mechanisms regulate fear extinction in adult and juvenile animals following exposure to stress^6,7^ and in response to OTR manipulations^5,8,9^.

OTR receptors are abundantly expressed in the mPFC^10–12^ and are known to play a role in the facilitation of extinction^9,13^ as well as in mediating social behavior^14–16^. In young juvenile animals, social behavior is predominantly characterized by social play behavior^17–19^. Lesions to either the mPFC or orbitofrontal cortex (OFC) in rats lead to changes in both adult social and juvenile play behaviors^20–22^.

Social behavior exists in male and female juvenile animals; female rats exhibit less anxiety-like behavior than their male conspecifics^23,24^. There have been inconsistent findings on the question of whether the brain OT system is sexually dimorphic in adult animals^25–27^. A previous study addressed age- and sex-dependent differences in OT plasma levels following exposure to stress, and reported that not only both male and female adults, but only juvenile females showed a significant increase in plasma OT levels following stress exposure^28^. These results may suggest that even prior to puberty, differences between males and females exist.

In this study, we use the paradigm that we developed for social interaction during the extinction of fear to address sex differences in juvenile animals and to explore if manipulations of systemic or prefrontal OT differently affect extinction and social interaction-induced facilitation of extinction.

## Materials and methods

### Animals

Experiments were performed on juvenile male and female Sprague Dawley rats aged 27 postnatal days (P27) and bred at the Haifa University animal facilities. The subjects were randomly selected from several litters to avoid the litter effect, and no more than two animals were taken from each litter. At postnatal day 20 (P20) the pups were weaned and housed in plexiglas cages and maintained on a free-feeding regimen with a 12-h light: 12-h dark schedule (7:00 AM to 7:00 PM). Sample size was determined according to our previous findings as sufficient to obtain statistically significant results^5^. In experiment 3, surgery of the juvenile was performed at P23, when the rats weighed 55–70 g. Animals were anesthetized using ketamine (65 mg per kg, intraperitoneally (i.p.)) and xylazine (7.5 mg per kg, i.p.), and restrained in a stereotactic apparatus^8,29–31^. The animals were implanted bilaterally with a stainless-steel guide cannula (23 gauge) aimed at the mPFC (aiming to the IL) (anteroposterior, +2.7 mm; lateral, ±0.6 mm; ventral, −3.8 mm; Supplementary Fig. S1)^31,32^. The cannulae were held in place with acrylic dental cement secured with two skull screws. A stylus was placed in the guide cannula to prevent clogging. The animals were allowed 3 days to recuperate before being subjected to experimental procedures^8,29,31^. The young rats have a much faster recovery rate than the adults. In numerous studies, 24 h are considered to be sufficient for juvenile’s recovery from cannula and electrode implantation surgery^33–36^. Thus our method of allowing for 3 days of recovery was designed to be the least traumatic for the juvenile animals. The procedures were performed in strict accordance with the University of Haifa ethical committee regulations and the US National Institutes of Health guidelines (NIH publication number 8023).

### Housing

All animals were housed in groups of four to five rats per cage in the animal room. Animals were randomly divided into “single” and “pairs” conditions. Animals that were assigned into the “pairs” condition were always taken from the same home cage. This pairing-assignment method, which is based on familiarity, is used to prevent aggressive behavior, as described in previous studies^5,37,38^. Due to the experimental setting of testing in either single or pairs, no blinding of the experimenter to the subject condition was possible.

### Contextual fear conditioning

Based on our previous conditioning and extinction protocols^5,29^, rats from all experimental groups were placed in a conditioning chamber with black methacrylate walls, a transparent front door, a top-view video camera, and a metal grid floor. For conditioning, each animal received three foot shocks delivered through the grids (0.6 mA lasting 0.5 s at 2-min intervals).

Rats were given 2 min for acclimatization to the context before delivery of the first shock and an additional 2 min after the last shock. In total, the whole conditioning session lasted about 8 min (for details^39,40^). The chamber was cleaned with 70% ethanol and dried with paper towels after each trial. All animals underwent fear conditioning separately.

### Retrieval session (Ret)

The retrieval session was performed at 24 h after conditioning. The conditioned rats were placed in a conditioning chamber for 5 min during which no shock was delivered. Afterward, based on freezing-rate data (comparable freezing levels ± 10%), they were divided into two groups: paired (pairs) and individuals (single). This allocation served to ensure even distribution of basal freezing rates.

### Extinction sessions (Ext1 and Ext2)

Rats underwent two consecutive 10-min extinction sessions separated by 24 h. The “pairs group” underwent extinction training together with data collected from both animals and rats in the “single group” underwent the same procedure but individually.

**Fig. 1:**
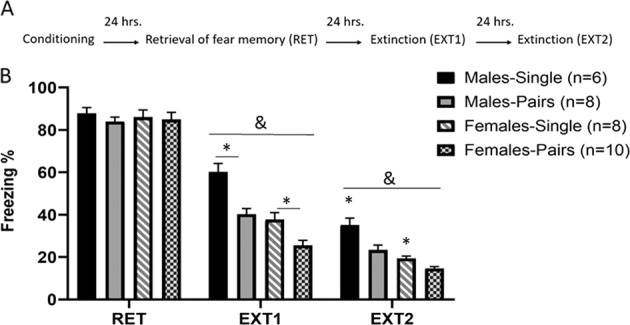
Enhanced extinction in male and female rats trained for extinction in pairs. **A** Schematic representation of the behavioral protocol. **B** Male and female rats were tested for the extinction of fear memory in pairs or individually (pairs/single). Freezing levels were similar between groups on the retrieval day (RET). ANOVA with repeated measures on freezing levels on extinction days (Ext1 and Ext2) showed significant effects of sex (^&^*P* < 0.001), with females extinguishing faster than males, and condition (^#^*P* < 0.001), with the pairs condition having higher extinction rates than the single condition.

**Fig. 2:**
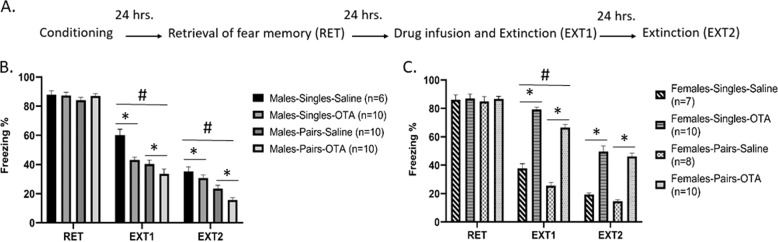
Oxytocin is involved in extinction and social-induced facilitation of extinction in females but not males. **A** Schematic representation of the behavioral protocol. **B**, **C** Male and female rats were tested for the extinction of fear memory in pairs or individually (pairs/single), and were systemically injected with saline or OT antagonist (OTA). Freezing levels were similar between groups on the retrieval day (RET). **B** Male freezing levels at Ext1 and Ext2 showed that the pairs condition extinguished faster than the single condition (^#^*P* < 0.001). Systemic inhibition of the OTR with an OTA resulted in reduced freezing levels compared to control, saline-treated animals, in both the pairs and single conditions (*P < 0.001). **C** In females, systemic inhibition of the OTR with an OTA resulted in impaired extinction in both pairs and single conditions (*P < 0.001). The Female-Pairs-Saline group was different from the Female-Single-Saline only at Ext1, but not Ext2 (^#^*P* < 0.001).

**Fig. 3:**
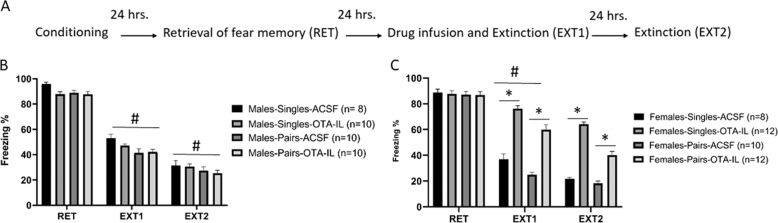
Prefrontal oxytocin is involved in extinction and social-induced facilitation of extinction in females but not males. **A** Schematic representation of the behavioral protocol. **B**, **C** Male and female rats were tested for the extinction of fear memory in pairs or individually (pairs/single), and were microinjected with ACSF or the OT antagonist (OTA). Freezing levels were similar between groups on the retrieval day (RET). **B** Male freezing levels at Ext1 and Ext2 showed that the pairs condition extinguished faster than the single condition (^#^*P* < 0.001). There was no effect of OTA in the IL- mPFC. **C** Females having OTA microinjected into the IL had significant impairment in extinction in both the pairs and single conditions. The Female-Pairs-Saline group was different from the Female-Single-Saline only at Ext1, but not Ext2 (^#^*P* < 0.001).

Schematic representations of the protocols are represented in Figs. 1–3A.

### Freezing

To record freezing, the data were analyzed with the high-sensitivity movement transducer of Ethovision software (Noldus), in which the animal’s movements were recorded by a color video camera for offline analysis. Motion and exploration were recorded and analyzed using a designated software component of Ethovision. To differentiate between the animals in the pairs condition, animals were marked with a Xylene-free, instant drying, and waterproof permanent marker. Freezing duration was defined as the overall time duration, in which the animal ceases all types of movement except for respiration^41–44^. Freezing duration was measured and averaged over all sessions. For the conditioning session, the first 2 min served for acclimatization to the chamber, during which the rat explored the cage. The last 2 min were used to estimate the success of the conditioning. For the extinction session, freezing was averaged over the whole session. Two criteria of exclusion were set: (1) animals that expressed immobility during the 2 min of acclimatization period prior to conditioning and (2) animals that expressed <50% of freezing at the retrieval test (Ret). Only two animals were excluded based on high freezing levels prior to conditioning.

### Biochemical analysis of oxytocin levels

To examine the levels of OT in the mPFC in males and females, naive animals were decapitated. The brains were quickly removed, frozen in liquid nitrogen to “snap freeze”, and stored at −80 °C until further processing. Bilateral micropunches of 1-mm diameter were taken from the IL-mPFC. The tissue micropunches were homogenized in ice-cold lysis buffer (100 Mm Tris pH 7.4, 150 mM NaCl, 1 mM EGTA, 1 mM EDTA, 1% Triton x-100, protease inhibitor, PMSF 1 mM), and then the homogenized samples were centrifuged at 14,000 rpm for 2 min. Only the superior fraction was collected, and OT levels were determined using an oxytocin ELISA kit (detection range: 15.6–1000 pg/mL; intra-assay variance for low concentration samples 12.6%; inter-assay variance for low concentration samples 20.9%; ab133050, Abcam).

### Drugs and administration

For microinjections, we used the OT receptor antagonist desGly-NH_2_,d(CH_2_)_5_[D-Tyr^2^,Thr^4^]OVT (kindly donated by Dr. M. Manning, University of Toledo, Ohio) dissolved in saline (0.9% NaCl) to a final concentration of 153 μmol/L (74.8 ng, this dose is based on the work of refs. ^5,45^).

For systemic injections, we used the OT antagonist hydrochloride (S)-2-amino-N-((1S, 2S, 4R)-7,7-dimethyl-1-((4-o-tolylpiperazin-1-ylsulfonyl)methyl) bicyclo[2.2.1] heptan-2-yl)-4 (methylsulfonyl) butanamide was (L-368,899) dissolved in saline to a final concentration of 1.5 mg/ml and injected i.p. at a dosage of 2 mg/kg. There is evidence demonstrating that peripherally administered L-368,889 crosses the blood–brain barrier^46^ and affects oxytocin-associated behaviors^47^. All control groups were injected with saline. The experimenter was blind to the injected drug.

All microinjections or injections were performed 30 min before the Ext1 session; this time point was chosen based on previous experimental findings^5,8,9^. For microinjections, the stylus was removed from the guide cannula, and a 28-mm gauge injection cannula was inserted. The injection cannula was connected via PE20 tubing to a Hamilton microsyringe driven by a microinfusion pump (Harvard Apparatus, USA). Microinjections were performed bilaterally using a volume of 0.5 µl per hemisphere delivered over 1 min. The injection cannula was left in position for an additional minute to minimize dragging of the injected solution along the injection tract.

### Statistics

On each of the testing days, freezing was averaged. Freezing is determined as the percentage of time spent freezing, and the results are expressed as means ± SEM. All data were analyzed using the Shapiro–Wilk test to examine sample distribution and analyzed using the *F* test to examine homoscedasticity. All normally distributed data were analyzed using mixed ANOVA. All the ANOVAs were followed by one-way ANOVA and Student’s *t* tests when needed. All tests were two-tailed, and a *P* value of <0.05 was considered statistically significant. All post-hoc comparisons were made using least significant difference (LSD) multiple comparison tests.

## Results

### Experiment 1: facilitation of extinction by social presence

The aim of this experiment was to examine whether social presence affects the memory of extinction similarly in juvenile males and females. To that end, animals underwent fear conditioning as singles, followed by retrieval session as singles or pairs. Animals were divided into four groups (male-singles: *n* = 6, male-pairs: *n* = 8; female-singles: *n* = 8; females-pair-: *n* = 10). There were no differences in the freezing levels during the retrieval test, between groups that were later divided into different conditions (*F*(1, 28) < 1, n.s.). No differences between males and females were observed (*F*(1, 28) < 1, n.s.; Fig. 1B), indicating comparable baseline freezing during the retrieval test.

ANOVA with repeated measures on freezing levels on extinction days (Ext1 and Ext2) showed significant effects of sex (males, females), and condition (singles, pairs) (*P* < 0.001 significance for both). However, without significant interaction of sex and condition (*F*(1, 28) = 2.24, n.s.). These results suggest that females exhibited lower freezing levels in both single and paired conditions than males and further show that extinguishing in pairs in both males and females facilitates extinction. A significant effect of the testing day was also found (*F*(1, 28) = 73.86, *P* < 0.001). A significant interaction for the testing day with the condition (*F*(1, 28) = 8.57, *P* < 0.01) was found but not with sex.

### Experiment 2: sex-dependent effect of systemic OT on single and paired extinction

The aim of this experiment was to examine whether the effect of social interaction can be modified by manipulating systemic oxytocin and the possible differences between juvenile males and females. To that end, animals underwent fear conditioning, followed by retrieval session as singles and divided into eight groups (male-single-saline: *n* = 6, male-single-OTA: *n* = 10; male-pairs-saline: *n* = 10, male-pairs-OTA: *n* = 10; female-single-saline: *n* = 7, female-single-OTA, *n* = 10, female-pairs-saline: *n* = 8, female-pairs-OTA: *n* = 10). There were no differences in the freezing levels during the retrieval test, between groups that were later divided into different conditions (*F*(1, 63) < 1, n.s.). No differences between males and females were observed (*F*(1, 63) < 1, n.s.; Fig. 2B), indicating a comparable baseline of freezing.

ANOVA with repeated measures on freezing levels on extinction days (Ext1 and Ext2) showed significant effects of drug (saline, OTA), sex (males, females), and condition (singles, pairs) (*P* < 0.001 significance in all). Furthermore, a significant interaction of drug and sex was found (*F*(1, 63) = 212, *P* < 0.001), but no significant interactions of condition and sex (ns) or condition and drug (n.s.). The testing day was found to be significant (*F*(1, 63) = 185, *P* < 0.001). A significant interaction was found for testing day, sex, and condition (*F*(1, 63) = 4.15, *P* < 0.005). All other interactions were not significant. A follow-up analysis by sex showed that in males, the significant main effects are condition (*F*(1, 32) = 38.0, *P* < 0.001), drug (*F*(1, 32) = 15.18, *P* < 0.001), and testing day (*F* (1, 32) = 150.07, *P* < 0.001), but without significant interactions. Specifically, animals in pairs had reduced freezing levels compared to singles. Interestingly, systemic inhibition of the OTR resulted in reduced freezing levels compared to the saline-treated animals. The lack of interaction between condition and drug suggests that no difference in the effects of drugs was observed in singles and pairs.

In females, significant effects of condition, drug, testing day, and interaction between testing day and drug were found, without other interactions. Follow-up showed that Ext1 was significantly affected by condition (*F*(1, 31) = 22.4, *P* < 0.05) and drug (*F*(1, 31) = 219.93, *P* < 0.001), but without significant interaction. However, the only significant effect on Ext2 was drug *(F*(1, 31) = 181.4, *P* < 0.001). The females in the pairs condition showed reduced freezing levels compared to the single condition only for Ext1, and females treated with the OTR antagonist showed enhanced freezing levels for Ext1 and Ext2.

These results suggest opposite effects of systemic inhibition of OTR in males and females regardless of whether tested in pairs or individually.

### Experiment 3: sex-dependent effect of prefrontal OT on single and paired extinction

We previously showed that in the adult animal, OT in the IL is important for the social-induced enhancement of extinction^5^. We thus examined the dependency of IL-OT in juvenile males and females. To that end, an OTR-selective antagonist (OTA) was microinfused into the IL-mPFC prior to extinction (Ex1) in singles and in pairs. The groups were assigned as following (male-single-saline: *n* = 8, male-single-OTA-IL: *n* = 10; male-pairs-saline: *n* = 10, male-pairs-OTA-IL: *n* = 10; female-singles-saline: *n* = 8, female-single-OTA-IL, *n* = 12, female-pairs-saline: *n* = 10, female-pairs-OTA-IL: *n* = 12).

No differences between the groups (*F*(1, 72) = 1.8; n.s.) were observed on the retrieval test, suggesting comparable freezing levels in all the groups.

ANOVA with repeated measures (sex (males, females), condition (single, pairs), drug (ASCF, OTA), testing days (Ext1, Ext2); (2X2X2×2)) showed significant effects of sex (*P* < 0.005), drug (*P* < 0.001), condition (*P* < 0.001), and interactions of drug, sex, and condition (*F*(1, 72) = 6.32, *P* < 0.05; Fig. 3B). Further, there was a significant interaction between testing day, drug, and condition (*F*(1, 72) = 12.29, *P* < 0.001), but without an interaction between testing day, drug, sex, and condition (*P* > 0.05).

A follow-up analysis showed that in males during Ext1 and Ext2, there was an effect of condition (*P* < 0.001) without an effect of drug (n.s.), suggesting that blocking OTR in the IL has no effect on extinction in males.

In contrast, a follow-up analysis for Ext1 and Ext2 in females showed interactions between condition, drug, and testing day (*F*(1, 38) = 5.3, *P* < 0.05). For Ext1, significant effects of condition (*F*(1, 38) = 42.47, *P* < 0.001] and of drug (*F*(1, 38) = 262.5, *P* < 0.0001) were found. However, no interaction was reported between the two variables (n.s.), suggesting differences between individuals and pairs and that the drug similarly affected the single and the pairs conditions.

For Ext2 however, a significant interaction between drug and condition (*F*(1, 38) = 8.43, *P* < 0.001) was found in addition to significant effects of drug (*F*(1, 38) = 368.3, *P* < 0.001] and condition (*F*(1, 38) = 57.66, *P* < 0.001). A follow-up analysis by drug showed no significant effect of condition (single or pairs) for the saline groups, while single and pairs significantly differed in the OTA groups (*t*(20) = 8.57, *P* < 0.001]. The single-OTA-IL maintained enhanced freezing levels compared to the pairs-OTA-IL (63.3 ± 1.8%; 40.3 ± 1.9, respectively), suggesting a beneficial effect of the pairs condition even under OTA-IL.

Together, these results suggest that inhibition of OTA in the IL has an effect on extinction only in females without affecting males, and that its effect in the pairs condition is moderate compared to the single condition.

### Differences in the OT levels in the mPFC of males and females

**Fig. 4:**
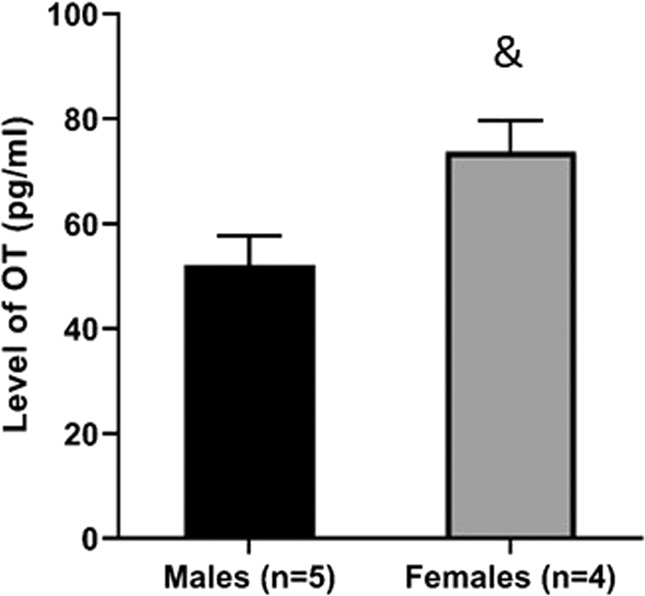
Juvenile males and females have different levels of prefrontal oxytocin. Juvenile males and females were decapitated, and OT levels were measured from punches from the mPFC. Independent *t* test showed that females have higher levels of OT in the mPFC (^&^*P* = 0.035).

The differential effects observed for males and females in response to OT manipulations motivated us to ask whether the two sexes have different baseline levels of OT in the mPFC. Accordingly, males and females were decapitated, and tissues from the IL-mPFC were taken for OT quantification [see Yaseen et al.^31^]. An independent *t* test showed that females have significantly higher OT levels than males (*t*(7) = 6.72, *P* = 0.035; males: 52.04 ± 5.63 pg/ml; females: 73.73 ± 5.9 pg/ml; Fig. 4).

## Discussion

We previously established a behavioral paradigm in which we showed that, in adult males, social presence facilitates the extinction of fear and that this social-induced facilitation is dependent on OT in the IL-mPFC^5^. In this study, we used the same paradigm and examined whether juvenile animals exhibit similar behavior while also addressing possible differences between juvenile female and male animals and the dependency of these differences on OT. Our results show (1) social interaction facilitates extinction in juvenile males and females, (2) systemic OTR antagonist impairs fear extinction in females in both single and pairs conditions, whereas it facilitates fear extinction in males, and (3) prefrontal OT is involved in extinction in females, but not in males, in both single and pairs conditions.

These results indicate that even at the juvenile stage, OTR inhibition exerts different effects in males and females.

### The effects of extinguishing in pairs on extinction of fear

Here and in our previous study, we tested the impact of extinguishing fear in pairs on the extinction. Our previous results in adult male rats showed that the social presence of two animals that previously underwent fear conditioning resulted in accelerated extinction rates compared to solitary extinction^5^. In this study, we replicate these findings in juvenile animals and report the beneficial contribution of social presence for fear extinction in both juvenile males and females; freezing levels were significantly lower in both sexes in the pair’s condition compared to the single condition. It is worthy of mentioning that in females, the facilitation in the pair’s condition is mainly reflected on Ext1 and less on Ext2 (Figs. 2 and 3); this is probably attributed to the floor effect that shows very low freezing levels in both conditions.

It could be argued as animals underwent conditioning as individuals, being tested in pairs may result in a perception of the conditioning context as a new one. We previously reported that in adult animals the context is not perceived as a new one as a reinstatement of fear even in the pairs condition showed high freezing levels^5^. However, as we did not conduct an experiment for reinstatement in this study, we suggest that also in the juvenile animal, the presence of another animal that underwent the same conditioning procedure can have a protective effect and help to reduce fear. Future studies should address and dissociate between social presence and exploratory behavior (for example, for an object) on fear reduction.

The reduction in freezing by social presence could be mediated by play behavior, as juvenile social play behavior is one of the earliest forms of non-mother-directed social behavior in rodents. This may imply that the way fear reduction is mediated by social presence may differ in juveniles and adults and may suggest that the social play induced by the presence of another animal may reduce context-induced fear. However, it should be noted that in this study, we did not examine other social behaviors, but we focused on freezing behavior.

### Sex differences in social interaction

Juvenile’s social play behavior is sexually dimorphic with males exhibiting higher levels compared to females. Males were reported to engage in more rough-and-tumble social play than females^48–51^. This sex difference was attributed largely to an increased rate of play initiation by males^48,50,52–55^. In this study, we do not observe differences between males and females, perhaps because the experimental setting does not allow for natural social play. Future studies should address the possible link between social play in juvenile rats and the reduction in fear responses. Interestingly, it was previously reported that females exhibit higher locomotor activity than males^56–58^. We dismiss the possibility that reduced freezing levels result from more locomotor activity in females since we did not observe differences in freezing levels between males and females during the retrieval or conditioning sessions. Further, it was reported that, juvenile female, but not male, animals show renewal of fear and spontaneous recovery following extinction training^59^, suggesting significant differences in extinction behavior between males and females^60^. Although we did not find qualitative differences between males and females or different locomotion activity during conditioning or retrieval, future studies should address whether differences in fear behaviors are influenced by baseline anxiety differences in males and females.

Importantly, dominance and kinship were not controlled for in this study. Dominance was found to be influential in socially transmitted fear conditioning memory in adult males^61^, and kinship was found to be of similar involvement in the adult females^62^. There are as yet no evidence of the role of these factors in the extinction learning of the juvenile male and female. Future studies should address these issues at the earlier stages of development.

### The role of oxytocin in extinction and the beneficial effect of social interaction

Another main finding of this study is the differential role of OT in mediating extinction in males and females. We addressed this by systemic and intra-IL-mPFC injections of OT antagonist. Although we expected to find differences in the pairs, but not singles, the results show opposite patterns in both sexes. Surprisingly, whereas systemic administration of an OT antagonist impaired extinction in females, it facilitated extinction in males, regardless of the testing condition. The systemic administration of OTA has previously been demonstrated to inhibit OTR within the brain for 6–18 h^63^. We speculate that the observed long-term effects of the drug on behavior are not related to its pharmacological properties per se, but rather due to an effect on memory processes that occurred immediately following the OTA administration and persisted for 48 h.

To the best of our knowledge, the majority of research addressing the effects of OT manipulations or behaviorally dependent release of OT has focused on adult male animals^28^. It was previously reported that exposure of male rats to novelty, forced swimming, or social defeat rapidly increases OT release into the blood but also within the PVN and/or SON and in other limbic brain regions, such as the central amygdala or septum^64,65^. It should be noted that we did not measure differences in OT release after the different conditions, we only addressed whether baseline differences in OT in the mPFC in males and females could mediate the observed differences in the effects of social interaction. Future studies should address how social interaction affects OT levels in the mPFC in juvenile males and females. Nonetheless, the results of the present report clearly show that not only OT levels are different in juvenile males and females, but using the same dose and time point, the effects of blocking the OTR are different in males and females. The results also suggest that regardless of the social condition of testing (pairs vs. single group), blocking the OTR results in similar effects within the same sex. Our current work did not consider dose-dependency, and it is plausible that different doses could elicit different effects. It was previously reported that in males, only adult animals show significant increases in plasma OT levels following exposure to restrain stress, while juveniles do not exhibit such an increase at all. Female adults have a higher baseline OT level and show a transient decrease in response to stress. Juvenile females have an increase in OT levels after stress and are, in this respect, surprisingly similar to the adult male^28^. Therefore, age and sex may determine the effects of OT on cognitive and emotional processes.

In addition, recent human studies have demonstrated that intranasal application of OT has a sex-dimorphic dose-dependent effect on the response to negative social stimuli. Specifically, males exhibit decreased activation of the amygdala in response to fearful faces when administered with an intermediate dose of OT. In contrast, females showed an increase in the same parameter even after the administration of lower doses of OT^66,67^. This implies that the application of exogenous OT may change the valence of social presence in a sex-dimorphic manner.

The mPFC, which is critically involved in mediating extinction of fear^68,69^, is especially sensitive to experiments involving both social and emotional tasks^70^. The mPFC has an abundance of OT receptors^71^, which are crucially involved in modulating social behavior in humans and nonhuman mammals^72,73^ and mediating anxiety response^74^. In addition, we and others previously showed that ICV microinfusion or direct microinfusion into the IL-mPFC of OT or its agonist could enhance fear extinction in adult animals^9,75^, while in juvenile animals, microinfusion of OT into the IL of mPFC had no effect on subsequent extinction^8^. In contrast, antagonizing OR in the IL of the adult male animal did not affect freezing in the Single condition but precluded the facilitatory effect of extinguishing in pairs^5^. In the juvenile male animals, similar treatment in both single and pairs conditions had no effect on subsequent freezing. Thus, blocking the OT system did not preclude the facilitatory effect of social interaction on extinction, suggesting that the enhancing effect of social interaction on extinction in juvenile males is not mediated through mPFC-OT, unlike the adult males^5^. These results join our recent reports in showing that the mechanisms mediating extinction in adult and juvenile animals may be distinctive^6–8,29^. The present findings provide strong evidence of sex difference, even before sexual maturation, in many biological processes and signaling and point to substantial differences between males and females^76^. This will open future research directions on sex differences in neurological and psychiatric disorders^76,77^.

## Supplementary information

Figure legend for Figure S1
