# Supplementary material for: Sex-dimorphic role of prefrontal oxytocin receptors in social-induced facilitation of extinction in juvenile rats
Source: Transl Psychiatry. 2020 Oct 19;10:356. doi: 10.1038/s41398-020-01040-9 (PMC7572379; doi:10.1038/s41398-020-01040-9)
Supplement: Supplementary file 1 — Figure legend for Figure S1 [file 41398_2020_1040_MOESM1_ESM.docx]

**Histology:** Upon completion of the behavioral experiments, animals were deeply anesthetized with urethane (40% urethane, 5% chloral hydrate in saline; 0.5 ml/100 g, i.p.), and Indian ink (0.5 µl) was microinfused into the IL and brains were stored at ‑80°C. Coronal sections of 30 µm were cut using a cryostat. Following Nissl staining, the locations of cannulae were examined under a light microscope. **Figure S**1 shows a schematic representation of the placement of cannulae in the IL (coronal view at position +3.20 and +2.70 mm anterior to bregma). Solid black circles indicate the locations. Animals that did not have the tips of their cannulae in the IL were excluded from analysis. Four animals were excluded.

**Fig. S1. Placement of cannulae in the IL-mPFC.**

The cannulae were implanted bilaterally with a stainless steel guide (23 gauge) aimed at the IL-mPFC [anteroposterior, +2.7 mm; lateral, ±0.6 mm; ventral, −3.8 mm relative to bregma].
